# Effectiveness of brace treatment for adolescent idiopathic scoliosis

**DOI:** 10.1186/1748-7161-10-S1-O62

**Published:** 2015-01-19

**Authors:** Toru Maruyama, Yosuke Kobayashi, Makoto Miura, Yusuke Nakao

**Affiliations:** 1 Saitama Medical Center, Saitama Medical University, Japan

## Objectives

Effectiveness of brace treatment for adolescent idiopathic scoliosis (AIS) was demonstrated by the BrAIST study in 2013. Objectives of this study were to analyze outcomes of our brace treatment to confirm the effectiveness of brace treatment for AIS and to clarify the factors affecting the results of the treatment.

## Materials and methods

According to the Scoliosis Research Society (SRS) AIS brace studies standardization criteria, patients with age 10 years or older, Risser 0 to II, less than 1 year post-menarche, curve magnitude 25 to 40 degrees before brace treatment and who received no prior treatment were included in the study. At skeletal maturity, the rate of the patients whose curve was stabilized, exceeded 45 degrees, and who were recommended or underwent surgery were investigated. Additionally, initial correction rate by the brace was calculated comparing pre-treatment Cobb angle and first in-brace Cobb angle. Factors that affected the results of the treatment were investigated.

## Results

A total of 33 patients (27 females and 6 males) could be followed-up until their skeletal maturity and included in the analysis. An average age was 11.9 years, average Cobb angle was 30.8 degrees, and Risser sign was 0 in 13 patients, I in 5 patients, and II in 15 patients before treatment. There were 13 thoracic curves, 14 thoracolumbar or lumbar curves, and 6 double curves. Initial correction rate by the brace was 53.8% for the total curves. In terms of curve pattern, 34.4% for thoracic curve, 73.9% for thoracolumbar or lumbar curve, and 48.8% for double curve.

After an average follow-up period of 33 months, 8 patients improved in more than 6 degrees, change of 17 patients were within 6 degrees, and 8 progressed in more than 6 degrees. Therefore, totally, 76% (25/33) of the curves were stabilized by the treatment. Four curves (12%) exceeded 45 degrees and one patient (3%) underwent surgery. Our results were better than the reported natural history. Factors that affected the results were hump degree (p = 0.0204) before treatment and initial correction rate by the brace (p = 0.0048).

## Conclusions

76% of the curve with AIS could be stabilized by brace treatment. The effectiveness of brace treatment for AIS was confirmed by our results. Factors that affected the results of the treatment were hump degrees before treatment and initial correction rate by the brace.

